# Case report: When encephalitis is not an infection: two cases of autoimmune GFAP astrocytopathy mimicking infectious encephalitis

**DOI:** 10.3389/fimmu.2026.1960742

**Published:** 2026-10-16

**Authors:** Gabriela Trojan, Ngoc Hoang Anh Than, Abisha Senthilkumar, Faith Emmanuel, Anna Moniuszko-Malinowska, Piotr Czupryna

**Affiliations:** 1 Department of Infectious Diseases and Neuroinfections, Medical University of Bialystok, Bialystok, Poland; 2 Medical University of Bialystok, Białystok, Poland

**Keywords:** autoimmune encephalitis, case report, diagnostic pitfall, GFAP astrocytopathy, GFAP-IgG, infectious encephalitis

## Abstract

**Background:**

Autoimmune glial fibrillary acidic protein (GFAP) astrocytopathy is a rare autoimmune inflammatory disease of the central nervous system that frequently mimics infectious meningoencephalitis. Because its clinical presentation, cerebrospinal fluid (CSF) findings, and inflammatory markers often resemble central nervous system infection, diagnosis may be delayed, postponing appropriate immunotherapy.

**Case Presentation:**

We describe two women with markedly different clinical presentations of GFAP astrocytopathy who were initially managed as suspected infectious meningoencephalitis. Both patients presented with inflammatory CSF findings, negative extensive microbiological investigations, and initial empirical antimicrobial treatment. In the first case, persistent aseptic meningitis with recurrent symptoms prompted autoimmune testing after repeated negative infectious work-up. In the second case, rapidly progressive encephalopathy requiring intensive care initially suggested severe infectious meningoencephalitis. In both patients, neuronal surface and paraneoplastic antibody testing was negative, whereas indirect immunofluorescence detected GFAP-IgG, supporting the diagnosis in the context of the clinical, CSF, neuroimaging, and microbiological findings. Both patients subsequently received corticosteroid treatment and experienced clinical improvement, although residual neurological symptoms persisted in the first patient.

**Conclusion:**

These cases highlight the broad clinical spectrum of GFAP astrocytopathy and its potential to closely mimic infectious central nervous system disease. Earlier recognition of GFAP astrocytopathy may facilitate timely consideration of immunotherapy and help reduce diagnostic delay and unnecessary antimicrobial exposure in patients with inflammatory meningoencephalitic presentations and negative infectious investigations.

## 1 Introduction

Autoimmune encephalitis (AIE) is a heterogeneous group of immune-mediated, inflammatory diseases that cause progressive brain inflammation. AIE is typically associated with autoantibodies against neuronal surface receptors, synaptic proteins, and intracellular antigens, though it may also be seronegative (1). AIE may be associated with underlying malignancy or, in some cases, preceding infections (2). Despite increasing recognition in recent years, these disorders remain underdiagnosed and underrecognized (3). Studies show that the incidence of AIE is comparable to that of infectious encephalitis (3) (4),. Rather than being considered only after exhaustive exclusion of infectious etiologies, autoimmune encephalitis should be included earlier in the differential diagnosis of encephalitis of unknown origin. This may facilitate timely antibody testing in selected patients and earlier consideration of appropriate immunotherapy.

The clinical presentation of AIE varies considerably according to the underlying subtype and may evolve over days to months. Common manifestations include headache, seizures, cognitive impairment, behavioral or psychiatric symptoms, and movement disorders (5–9). This clinical heterogeneity may result in initial misdiagnosis and delayed recognition of autoimmune disease (10–14). A notable example of diagnostic difficulty is Glial Fibrillary Acidic Protein (GFAP) astrocytopathy, first described in 2016 (15, 16). GFAP astrocytopathy is an inflammatory disorder of the central nervous system associated with IgG autoantibodies against the intracellular astrocytic protein GFAP, with antibody detection in CSF considered particularly diagnostically informative (17). Although the exact immunopathogenic mechanisms remain incompletely understood, accumulating evidence suggests that cellular immune mechanisms, including T-cell-mediated responses, may contribute to CNS inflammation and tissue injury (18). The clinical spectrum is broad and includes encephalitis, meningoencephalitis, meningoencephalomyelitis, and myelitis (19, 20). A characteristic neuroimaging finding is linear perivascular radial enhancement, although this pattern is not present in all patients and neuroimaging abnormalities may be absent or nonspecific (21). Early recognition is clinically important because corticosteroid treatment is associated with favorable outcomes in most reported patients (22, 23).

The diagnosis of GFAP astrocytopathy requires integration of the clinical phenotype, evidence of CNS inflammation, GFAP-IgG findings, neuroimaging, and exclusion of alternative diagnoses. No universally accepted formal diagnostic criteria defining definite, probable, and possible GFAP astrocytopathy have been established (24–26). Brain MRI and CSF analysis may provide evidence of CNS inflammation but are not specific for autoimmune disease (27). GFAP-IgG testing therefore provides an important component of the diagnostic evaluation, but antibody findings should be interpreted in the context of the clinical presentation, CSF findings, neuroimaging, and exclusion of infectious, neoplastic, and other autoimmune disorders (28). Importantly, GFAP-IgG may be detected in both CSF and serum, although CSF testing is considered more specific for GFAP astrocytopathy (29). The diagnostic challenge is particularly relevant in patients whose presentation closely resembles infectious meningoencephalitis but whose extensive microbiological investigations remain negative. In such cases, the absence of characteristic neuroimaging findings should not necessarily exclude GFAP astrocytopathy. We report two patients with inflammatory meningoencephalitic presentations who initially underwent evaluation and treatment for suspected infection but were subsequently found to have GFAP-IgG. The cases illustrate two diagnostically distinct presentations: persistent aseptic meningitis with GFAP-IgG detected in both CSF and serum despite normal brain MRI, and rapidly progressive encephalopathy with serum GFAP-IgG positivity but negative CSF testing and nonspecific meningeal enhancement. These cases highlight the importance of considering GFAP astrocytopathy in patients with inflammatory CNS syndromes that remain unexplained despite extensive infectious investigations.

## 2 Case descriptions

### 2.1 Case 1

A 36-year-old woman presented with a 5-day history of low-grade fever and severe stabbing headaches. Her past medical history was unremarkable. Neurological examination was unremarkable except for marked meningeal signs. A computed tomography (CT) scan of the head revealed no abnormalities. CSF analysis demonstrated inflammatory findings with lymphocytic pleocytosis (183 cells/µL; 97.8% lymphocytes) and elevated protein concentration (83.3 mg/dL). CSF polymerase chain reaction (PCR) testing did not identify an infectious etiology. Serological testing for tick-borne infections, including Borrelia burgdorferi, was negative. Based on the clinical presentation and initial laboratory findings, the patient was initially diagnosed with lymphocytic meningitis syndrome. She received intravenous dexamethasone (Dexaven) for 9 days, starting at 8 mg/day for 2 days, followed by 12 mg/day for 3 days, then tapered to 8 mg/day for 3 days and 4 mg/day on the final day, together with symptomatic treatment. A follow-up lumbar puncture showed partial improvement, with a WBC count of 130 cells/µL and protein concentration of 46.1 mg/dL. After 12 days of hospitalization, she was discharged in good condition on 11 March 2026.

After discharge, the patient continued to experience headache and photophobia, resulting in two visits to the Emergency Department. Head CT performed during these visits showed no abnormalities. On 14 March 2026, three days after the initial discharge, she was readmitted because of severe orthostatic headache accompanied by upper-limb tremor and dizziness. Neurological examination showed neck stiffness and a positive Kernig sign. Brain MRI showed no abnormalities. Contrast-enhanced spinal MRI demonstrated disc herniation at L3–L4 and L4–L5, with possible nerve root compression. CSF analysis showed persistent lymphocytic pleocytosis (178 cells/µL; 97.7% lymphocytes) with a normal protein concentration (36.6 mg/dL).

Samples were sent to the Laboratory of Neuroimmunology, II Department of Neurology, Institute of Psychiatry and Neurology in Warsaw for autoimmune antibody testing. Neuronal surface antibodies were tested using indirect immunofluorescence assay (IIFT) on transfected EU90 cells at a serum/CSF dilution of 1:10. Antibodies against NMDAR, AMPAR1/2, DPPX, LGI1, CASPR2, and GABABR were not detected. GFAP-IgG was also tested using IIFT on transfected EU90 cells at a dilution of 1:10 and was detected in both CSF and serum. In addition, the patient underwent extensive infectious and immunological investigations, including HIV testing, testing for Borrelia burgdorferi and tick-borne encephalitis, Treponema pallidum serology, amphiphysin antibodies, lymphocyte immunophenotyping, and repeated molecular testing for infectious pathogens (**Table 1**).

**Table 1:** Infectious disease investigations performed in the two patients.

Investigation	Case 1	Case 2
**HIV**	negative, repeated	negative
**Borrelia**	negative	negative
**TBE**	negative	negative
***T. pallidum***	TPHA/VDRL negative	—
**CSF multiplex PCR**	negative	negative
**Broad real-time PCR panel**	negative	negative
**CSF culture**	—	negative
**Blood cultures**	—	negative
**Respiratory cultures**	—	negative
***Toxoplasma***	—	negative

Infectious disease investigations performed in the two patients CSF multiplex PCR testing included E. coli, H. influenzae, L. monocytogenes, N. meningitidis, S. agalactiae, HSV-1, HSV-2, HHV-6, human parechovirus, VZV, and Cryptococcus neoformans/gattii. Broad real-time PCR panel covering CMV, EBV, HSV-1, HSV-2, HHV-6, HHV-7, VZV, adenovirus, hepatitis E virus, human parechovirus, measles virus, parvovirus B19, H. influenzae, N. meningitidis, S. pneumoniae, group B Streptococcus, L. monocytogenes, and E. coli.

Bold formatting highlights the infectious agents investigated and the diagnostic tests performed.

During hospitalization, the patient received a 5-day course of acyclovir because of lip lesions suspicious for herpes simplex infection and an 8-day course of doxycycline, together with symptomatic treatment. Because of persistent hamstring muscle contractures initially considered to contribute to the positive Kernig sign, the patient received physiotherapy. Subsequent assessment suggested that the limitation of knee extension was attributable to muscular contractures rather than meningeal irritation. After 20 days, her clinical condition improved, and she was discharged in good condition on 3 April 2026. Prior to discharge, she underwent otolaryngological, psychiatric, and neurological consultations.

Following confirmation of the diagnosis, oral prednisone was initiated at 60 mg/day (40 mg in the morning and 20 mg at noon) for 4 days, followed by 50 mg/day (30 mg in the morning and 20 mg at noon). The subsequent tapering regimen could not be verified because outpatient neurology records were unavailable. The patient was referred for ongoing neurological care.

During a telephone follow-up approximately one month after discharge, the patient reported partial clinical improvement; however, upper-limb tremor and generalized weakness persisted. She remained under regular neurological follow-up and continued corticosteroid treatment. The current dosing regimen could not be verified because neurology records were not available for review.

### 2.2 Case 2

On 2 January 2026, a 57-year-old woman with a medical history of migraine with aura and depression was admitted to the Emergency Department (ED) and was subsequently transferred to the intensive care unit (ICU) on the same day with acute neuropsychiatric symptoms and suspected stroke following a sudden episode of behavioural disturbance. According to her husband, she became confused, agitated, and delirious, spoke in an altered voice, and exhibited movements described as “wiggling out”.

Initial CT angiography of the head and neck, followed by non-contrast CT of the head, demonstrated no abnormalities. Laboratory investigations revealed elevated inflammatory markers (CRP 34.5 mg/L), prompting lumbar puncture. CSF analysis demonstrated lymphocytic pleocytosis of 99 cells/µL (88% lymphocytes) and an elevated protein concentration of 139 mg/dL. No infectious etiology was identified at this stage.

On neurological consultation, the patient was conscious but uncooperative. Pupillary reflexes were preserved, with no focal motor or sensory deficits. Due to severe agitation and risk of self-harm, sedation and physical restraints were required. A neuroinfectious disease consultation was requested because of the CSF pleocytosis, with a recommendation for brain MRI with contrast. Empirical treatment with acyclovir, ceftriaxone, ampicillin, and vancomycin was initiated pending identification of the underlying etiology.

Despite empirical therapy, the patient continued to deteriorate and developed acute respiratory failure requiring intensive care and mechanical ventilation, as well as enteral/parenteral nutritional support. She remained in the intensive care unit (ICU) from 2 January to 21 January 2026. Brain MRI performed on day 6 showed slight segmental meningeal thickening with moderate contrast enhancement and no other significant abnormalities.

Worsening inflammatory markers, with CRP increasing from 34.5 mg/L to 167 mg/L, prompted escalation of antimicrobial therapy to meropenem and metronidazole. Acyclovir was continued at the same dose. A comprehensive CSF microbiological panel was requested to further investigate potential infectious causes (**Table 1**).

During the ICU stay, prolonged respiratory insufficiency necessitated tracheostomy. On day 14, a Protex tracheostomy tube was placed under general anesthesia. Repeat CSF analysis performed on the same day showed partial improvement, with pleocytosis decreasing to 81 cells/µL and protein concentration to 60 mg/dL. Comprehensive microbiological and pathomorphological investigations excluded bacterial and viral CNS infection. CSF and serum samples were sent for autoimmune encephalitis antibody testing, including NMDAR, AMPAR1/2, DPPX, LGI1, CASPR2, and GABAB antibodies. Treatment was continued while awaiting the results.

The patient’s condition gradually improved. Mechanical ventilation was discontinued and replaced with passive oxygen therapy via the tracheostomy tube. By day 19, the patient was conscious and able to follow commands, although muscle strength was reduced in all four limbs. During the subsequent ICU hospitalization, she developed loose stools and upper abdominal discomfort; testing for Clostridioides difficile infection was negative. Laboratory abnormalities, including anemia, elevated inflammatory markers, and elevated liver enzymes, gradually normalized with treatment.

Repeat CSF testing demonstrated further improvement, with pleocytosis decreasing to 26 cells/µL and protein concentration to 32.1 mg/dL. Testing for antibodies against neuronal surface antigens in CSF and serum was negative. GFAP-IgG was tested in both CSF and serum using indirect immunofluorescence assay (IIFT) on transfected EU90 cells at a dilution of 1:10. The serum sample demonstrated a characteristic α-GFAP staining pattern and was interpreted as positive/probable α-GFAP antibody positivity by the reference laboratory, whereas the CSF sample was negative for GFAP-IgG. The characteristic staining pattern was interpreted and confirmed by experienced personnel at the Laboratory of Neuroimmunology, II Department of Neurology, Institute of Psychiatry and Neurology, Sobieskiego 9, Warsaw, Poland. Antibody titers were not available in the medical records.

On 21 January 2026, after completion of the ICU stay, the patient was transferred to the Infectious Diseases Department, where she remained hospitalized until 17 February 2026. During her hospitalization she received intravenous dexamethasone (Dexaven) for 19 days, starting at 8 mg/day on the first day, followed by 12 mg/day for 9 days, then 8 mg/day for 2 days, and 4 mg/day for the final 4 days, together with symptomatic treatment.

At the time of transfer, the patient remained in serious condition but was alert and oriented. Due to the tracheostomy, she communicated using gestures and writing. To maintain an oxygen saturation of approximately 95%, she remained on passive oxygen therapy. Her respiratory status progressively improved, with resolution of swallowing dysfunction and reduced need for bronchial suctioning. An initial attempt at tracheostomy tube removal was unsuccessful and resulted in acute respiratory deterioration with severe desaturation to 34%. Following further pulmonary and otolaryngological management, gradual respiratory recovery was achieved, and the tracheostomy tube was ultimately removed without complications.

The patient underwent intensive physiotherapy and was discharged home on 17 February 2026 with referral for neurological rehabilitation and outpatient neurological follow-up. Because of limited mobility, prophylactic low-molecular-weight heparin was prescribed.

During follow-up at the outpatient infectious diseases clinic, the patient reported complete resolution of symptoms. Neurological examination revealed no significant abnormalities, indicating a favorable clinical outcome. According to the available medical information, on 14 May 2026 the patient was diagnosed with bilateral coxarthrosis secondary to hip dysplasia, with osteoarthritis of the left hip. This orthopedic condition was unrelated to the neurological disease described in this report. Further details regarding its diagnostic work-up and management were not available for review.

The main clinical, cerebrospinal fluid, neuroimaging, immunological, therapeutic, and outcome characteristics of both patients are summarized in **Table 2**.

**Table 2:** Comparative clinical, laboratory, neuroimaging, and immunological characteristics of the two patients with suspected GFAP astrocytopathy.

Feature	Case 1	Case 2
Age/sex	36-year-old woman	57-year-old woman
Initial presentation	Fever, severe headache, photophobia, meningeal signs	Acute behavioral disturbance, confusion, agitation, altered voice, movement abnormalities
Initial working diagnosis	Lymphocytic meningitis	Infectious meningoencephalitis
CSF WBC	183/µL → 178/µL	99/µL → 81/µL → 26/µL
CSF differential	Lymphocytic	Lymphocytic
CSF protein	83.3 mg/dL → 36.6 mg/dL	139 mg/dL → 60 mg/dL → 32.1 mg/dL
CSF glucose	55 mg/dL	58 mg/dL
MRI	No significant abnormalities	Meningeal enhancement
Infectious work-up	Negative	Negative
Neuronal surface antibodies	NMDA, AMPA1, AMPA2, DPPX, LGI1, CASPR2, GABAB Negative	NMDA, AMPA1, AMPA2, DPPX, LGI1, CASPR2, GABAB Negative
Steroid treatment	Dexaven i.v., encorton p.o.	Dexaven i.v.
Other treatment	Analgesics, sedative medication, muscle relaxant (tizanidine), and symptomatic/supportive therapy	Antibiotic therapy (levofloxacin), antifungal therapy (fluconazole), analgesics, bronchodilator therapy (ipratropium bromide/fenoterol), and supportive hepatoprotective treatment
Malignancy screening	tumor markers (S-100, CA-125, HE4, CA72-4, CYFRA 21-1, AFP, CA19-9, and CEA)	tumor markers (S-100, CA-125, HE4, CA72-4, CYFRA 21-1, AFP, CA19-9, and CEA)
Outcome	Partial improvement; residual tremor/weakness	Complete clinical recovery
Follow-up	1 months	1 month
GFAP-IgG CSF	Positive	Negative
GFAP-IgG serum	Positive	Positive
Diagnostic certainty	CSF + serum supported	Probable, serum-based

## Discussion

GFAP-IgG is considered an important disease-associated biomarker of autoimmune GFAP astrocytopathy; however, because GFAP is an intracellular astrocytic protein, the direct pathogenic role of GFAP-IgG remains uncertain. Current evidence suggests that cellular immune mechanisms, including T-cell-mediated responses, may contribute substantially to the inflammatory CNS injury observed in this disorder.

Our cases illustrate why GFAP astrocytopathy remains one of the challenging mimics of infectious meningoencephalitis in clinical practice. First described in 2016, GFAP astrocytopathy is an autoimmune inflammatory disorder of the central nervous system with a broad and heterogeneous clinical spectrum (30).

Given that GFAP is an intracellular antigen, current evidence suggests that the underlying inflammatory process may involve predominantly T-cell-mediated mechanisms (18, 31). This may contribute to a broad clinical spectrum extending from optic nerve involvement to spinal cord disease (23). This clinical heterogeneity, together with the frequent occurrence of nonspecific inflammatory findings, may result in initial consideration of infectious etiologies and delay recognition of the underlying autoimmune disorder (11, 32). Because these patients often present with clinical phenotypes that overlap with other neurological syndromes, initial misdiagnosis is common, leading to delays in the initiation of targeted immunotherapy (12, 32). The disease typically follows an acute or subacute onset, with patients often presenting with non-specific, flu-like prodromal symptoms such as fever, malaise, and rhinorrhea (33). As the inflammatory process intensifies, these symptoms subsequently evolve into more severe neurological manifestations, including meningitis, encephalitis, and myelitis, or overlapping syndromes like meningoencephalomyelitis.

A meta-analysis by Hagborm et al. (2024) demonstrated the broad clinical and radiological spectrum of GFAP astrocytopathy. A preceding infectious prodrome was reported in approximately 45% of patients, most commonly involving the upper respiratory or gastrointestinal tract. The most frequent clinical phenotypes were meningoencephalomyelitis (32%), meningoencephalitis (24%), and encephalitis (12%), while fever, movement disturbances, and headache were among the most common symptoms (34). MRI abnormalities were also heterogeneous, with T2/FLAIR hyperintensities and perivascular or leptomeningeal enhancement reported in a substantial proportion of patients, while spinal cord involvement was frequently observed (34). CSF typically demonstrates lymphocytic pleocytosis and elevated protein concentrations, often without an identifiable infectious pathogen (35, 36).

Other neural autoantibodies may coexist with GFAP-IgG, including NMDAR-IgG, AQP4-IgG, and MOG-IgG (34). The coexistence of GFAP-IgG with MOG-IgG is of particular clinical relevance, as MOG antibody-associated disease (MOGAD) may also present with encephalitic manifestations and can therefore overlap clinically with GFAP astrocytopathy. MOGAD encompasses a broad spectrum of inflammatory demyelinating presentations, including acute disseminated encephalomyelitis, optic neuritis, myelitis, and cortical or brainstem encephalitic syndromes. Therefore, MOG-IgG testing may be considered in patients with compatible clinical or radiological features, particularly when the presentation does not fully conform to a typical GFAP-associated syndrome (37, 38).

The two patients described in the present report illustrate diagnostically distinct manifestations of this spectrum. In Case 1, the clinical presentation was dominated by fever, severe headache, and prominent meningeal signs, with persistent lymphocytic CSF pleocytosis despite a normal brain MRI. In Case 2, the initial presentation was characterized by acute neuropsychiatric symptoms, movement abnormalities, and severe encephalopathy requiring intensive care and mechanical ventilation. Brain MRI in Case 2 demonstrated slight segmental meningeal thickening with moderate contrast enhancement, but no characteristic radial perivascular enhancement or other specific abnormalities. Thus, neither patient demonstrated the classic neuroimaging pattern commonly associated with GFAP astrocytopathy.

Both patients had inflammatory CSF findings, including lymphocytic pleocytosis and elevated protein concentrations, together with extensive negative infectious investigations. Their initial presentations therefore closely resembled infectious meningoencephalitis and appropriately prompted microbiological evaluation and empirical antimicrobial or antiviral treatment. In Case 1, the initial working diagnosis was lymphocytic meningitis syndrome, based on the clinical presentation and inflammatory CSF profile. In Case 2, the rapid clinical deterioration and systemic inflammatory response further supported the initial concern for an infectious CNS process. However, repeated and comprehensive microbiological investigations did not identify a bacterial or viral pathogen in either patient.

The diagnostic interpretation of the GFAP-IgG results differed between the two cases. In Case 1, α-GFAP antibodies were detected in both CSF and serum using indirect immunofluorescence on transfected EU90 cells, supporting the diagnosis in the context of the compatible inflammatory CNS phenotype and extensive exclusion of alternative causes. In Case 2, GFAP-IgG was tested in both serum and CSF using the same methodology. A characteristic α-GFAP staining pattern was detected in serum and interpreted as positive/probable α-GFAP antibody positivity by the reference laboratory, whereas the CSF sample was negative. Thus, the diagnostic evidence was stronger in Case 1, in which GFAP-IgG was detected in CSF and serum, whereas Case 2 represents a less definitive presentation in which serum GFAP-IgG positivity was considered together with the inflammatory clinical phenotype, CSF pleocytosis and elevated protein concentration, meningeal enhancement on MRI, and extensive exclusion of infectious and other alternative diagnoses.

The discrepancy between serum and CSF GFAP-IgG findings in Case 2 may reflect differences in antibody detectability between compartments; however, the possibility that CSF antibody levels were below the detection threshold at the time of sampling cannot be excluded. This finding further emphasizes that serum GFAP-IgG should not be interpreted in isolation but rather in conjunction with the clinical phenotype, CSF findings, neuroimaging, and exclusion of alternative diagnoses.

The diagnosis of autoimmune GFAP astrocytopathy therefore remains challenging, particularly in patients presenting with an inflammatory meningoencephalitic syndrome and nonspecific or normal neuroimaging. Although exclusion of infection remains an essential component of the diagnostic evaluation, waiting until all microbiological investigations have been exhausted before considering autoimmune etiologies may contribute to diagnostic delay. In patients with persistent aseptic meningitis, meningoencephalitis, or encephalitis, particularly when repeated microbiological studies remain negative and the clinical course is not adequately explained by infection, autoimmune testing should be considered earlier.

GFAP-IgG testing provides an important component of this diagnostic evaluation. CSF testing is generally considered more specific for GFAP astrocytopathy than serum testing (39). A two-step laboratory approach combining tissue-based immunofluorescence with confirmatory cell-based testing has been proposed to improve the specificity of GFAP-IgG detection (32). Nevertheless, antibody results should always be interpreted in the context of the complete clinical picture rather than being considered diagnostic in isolation. This is particularly relevant to serum-only positivity, as illustrated by Case 2.

Both patients initially underwent extensive investigations for infectious causes and received empirical antimicrobial or antiviral treatment. In Case 1, clinical persistence and subsequent recurrence of symptoms despite initial treatment prompted further diagnostic evaluation. In Case 2, progressive clinical deterioration despite empirical antimicrobial and antiviral therapy led to intensive care management and continued diagnostic investigation. These courses illustrate the practical difficulty of distinguishing autoimmune inflammatory CNS disease from infection during the early stages of presentation. Earlier consideration of autoimmune etiologies in patients with persistent inflammatory CNS syndromes and repeatedly negative microbiological investigations may facilitate more timely GFAP-IgG testing and reduce unnecessary antimicrobial exposure.

Patients with GFAP astrocytopathy generally respond favorably to immunotherapy, particularly high-dose intravenous corticosteroids during the acute phase, although incomplete recovery and fatal outcomes have also been reported (34–36). Additional immunotherapies, including intravenous immunoglobulin, plasma exchange, rituximab, and cyclophosphamide, have been used in selected patients (40). Relapses have also been reported, particularly during corticosteroid tapering, supporting the need for clinical follow-up and consideration of maintenance immunotherapy in selected cases (35).

Both of our patients received systemic corticosteroid treatment during their clinical courses, although the treatment regimens and timing differed between the cases. In Case 1, dexamethasone was administered during the initial hospitalization, followed by oral prednisone after GFAP-IgG results became available. In Case 2, corticosteroid treatment including hydrocortisone was administered during hospitalization; however, the exact dose, duration, and timing could not be reliably reconstructed from the available medical records. Both patients subsequently demonstrated gradual clinical improvement, but the retrospective documentation does not allow a direct causal relationship between corticosteroid administration and clinical improvement to be established. In Case 1, residual upper-limb tremor and generalized weakness persisted at approximately one-month follow-up, although the patient continued to improve clinically.

The discrepancy between serum and CSF GFAP-IgG findings in Case 2 may reflect differences in antibody detectability between compartments; however, the possibility that CSF antibody levels were below the detection threshold at the time of sampling cannot be excluded.

## Conclusion

Autoimmune GFAP astrocytopathy should be considered in patients presenting with persistent aseptic meningitis, meningoencephalitis, or encephalitis of unknown etiology, particularly when extensive microbiological investigations remain negative and the clinical response to empirical antimicrobial therapy is limited. Our two cases illustrate the broad clinical spectrum of this disorder and highlight its potential to closely mimic infectious central nervous system disease. Early recognition of GFAP astrocytopathy may facilitate timely consideration of immunotherapy and help reduce diagnostic delay and unnecessary antimicrobial exposure in patients with inflammatory meningoencephalitic presentations and negative infectious investigations.

## Data Availability

The datasets used and analyzed during the current study are available from the corresponding author on reasonable request.
